# Pulmonary disease caused by a newly identified mycobacterium: *Mycolicibacterium toneyamachuris*: a case report

**DOI:** 10.1186/s12879-020-05626-y

**Published:** 2020-11-25

**Authors:** Tomoki Kuge, Kiyoharu Fukushima, Yuki Matsumoto, Yuko Abe, Eri Akiba, Kako Haduki, Haruko Saito, Tadayoshi Nitta, Akira Kawano, Takahiro Kawasaki, Takanori Matsuki, Hiroyuki Kagawa, Daisuke Motooka, Kazuyuki Tsujino, Mari Miki, Keisuke Miki, Seigo Kitada, Shota Nakamura, Tetsuya Iida, Hiroshi Kida

**Affiliations:** 1 Department of Respiratory Medicine, National Hospital Organization Osaka Toneyama Medical Center, 5-1-1 Toneyama Toyonaka, Osaka, Japan; 2 Department of Respiratory Medicine and Clinical Immunology, Osaka University Graduate School of Medicine, 2-2 Yamadaoka, Suita, Osaka, Japan; 3 Laboratory of Host Defense, World Premier Institute Immunology Frontier Research Center (WPI-IFReC), Osaka University, 3-1 Yamadaoka, Suita, Osaka, Japan; 4 Department of Infection Metagenomics, Genome Information Research Center, Research Institute for Microbial Diseases (RIMD), Osaka University, 3-1 Yamadaoka, Suita, Osaka, Japan; 5 Department of Respiratory Medicine, Yao Tokushukai General Hospital, 1-17 Wakakusa-cho, Yao, Osaka, Japan

**Keywords:** Non-tuberculous mycobacteria, *Mycolicibacterium toneyamachuris*, *Mycolicibacterium mucogenicum*, Rapid growing mycobacteria

## Abstract

**Background:**

Non-tuberculous mycobacterial pulmonary disease (NTM-PD) is becoming a significant health burden. Recent advances in analysis techniques have allowed the accurate identification of previously unknown NTM species. Here, we report a case of NTM-PD caused by a newly identified mycobacteria in an immunocompetent patient.

**Case presentation:**

A 44-year-old woman was referred to our hospital due to the frequent aggravation of her chronic respiratory symptoms, with NTM-PD-compatible computed tomography findings. Unidentified mycobacterium was repeatedly isolated from respiratory specimens and we diagnosed her as NTM-PD of unidentified mycobacterium. Subsequent whole-genome analysis revealed that the unidentified mycobacterium was a novel mycobacterium genetically close to *Mycolicibacterium mucogenicum*. We started combination therapy with clarithromycin, moxifloxacin, amikacin, and imipenem/cilastatin, referring to drug sensitivity test results and observed its effect on *M. mucogenicum* infection. Her symptoms and radiological findings improved significantly.

**Conclusion:**

We report a case of NTM-PD caused by a newly identified mycobacteria, *Mycolicibacterium toneyamachuris*, genetically close to *M. mucogenicum*. This pathogenic mycobacterium showed different characteristics from *M. mucogenicum* about clinical presentation and drug sensitivity. The clinical application of genomic sequencing will advance the identification and classification of pathogenic NTM species, and enhance our understanding of mycobacterial diseases.

## Background

The prevalence of non-tuberculous mycobacterial pulmonary disease (NTM-PD) is increasing worldwide and is becoming a significant health burden [1]. Recent advances in analysis techniques have allowed the identification of previously unknown NTM species [2–5].

Here, we report a case of NTM-PD caused by a newly identified mycobacteria genetically close to *Mycolicibacterium mucogenicum*. This novel mycobacterium caused chronic and progressive pulmonary disease in an immunocompetent patient. Furthermore, drug susceptibility and clinical presentation were different from those reported for *M. mucogenicum* infections [6].

## Case presentation

**Fig. 1:**
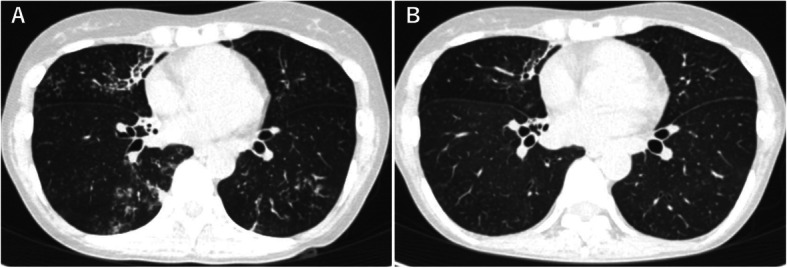
Chest CT before treatment shows small centrilobular nodules in the middle and lower lobes and slight bronchiectasis with a consolidation in the middle lobe (**a**). After 2 months treatment, small centrilobular nodules almost vanished (**b**)

**Fig. 2:**
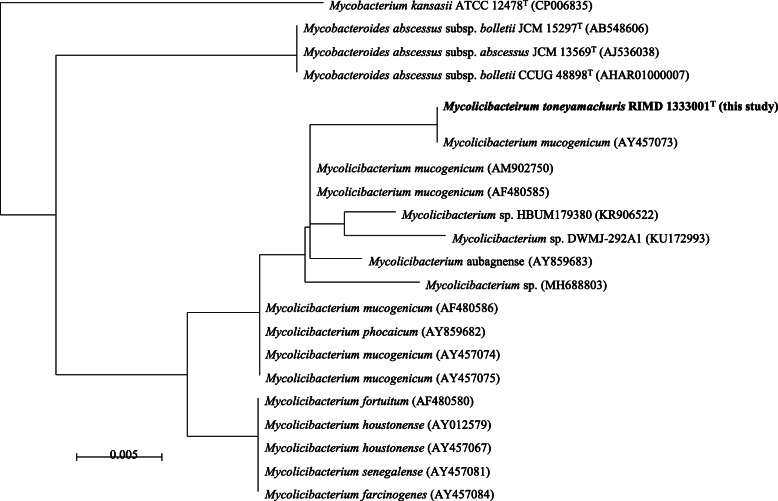
Approximately maximum likelihood phylogenetic tree using 16S rRNA sequences. Sequences were obtained from SILVA database release 138 as SSU Ref NR 99 sequences, which were showing larger than 98.7% of identity to the strain XXX T or derived from the type strains of Mycobacterium kansasii and Mycobacteroides abscessus

A 44-year-old woman, never smoker, was referred to our hospital 18 months ago due to chronic productive cough. She had asthma treated with inhalation therapy and allergic rhinitis. Chest computed tomography showed centrilobular nodules and bronchiectasis in the middle lobe and in the bilateral lower lobes (Fig. 1). Despite treatment with erythromycin and expectorants, her chronic respiratory symptoms worsened. Subsequently, a rapid growing mycobacterium (RGM), strain TY81, was cultured repeatedly from her sputum; however, its species/subspecies could not be identified by conventional methods such as AccuProbe (Gen-Probe Inc., San Diego, CA, USA), COBAS AMPLICOR (Roche Diagnostic, Tokyo, Japan), or DNA-DNA hybridization assay (Kyokuto Pharmaceutical Industrial, Tokyo, Japan). Therefore, we diagnosed her as NTM-PD of unidentifiable mycobacteria in accordance with the American Thoracic Society/Infectious Diseases Society of America (ATS/IDSA) criteria for the diagnosis of NTM-PD [7]. Multilocus sequence typing [8] revealed that the unidentified mycobacterium was genetically close to *M. mucogenicum* (Fig. 2).

We performed a whole-genome analysis of TY81 and obtained the complete genome sequence (AP023362-AP023365). The DNA G + C content of the type strain was 67.18 mol%. The mean nucleotide identity (ANI) to *M. mucogenicum* was 93.3% and was the maximum value obtained among the type strains of 175 NTM species (Table 1). Phylogenetic analysis using the 16S rRNA sequence suggested that TY81 was closely related to *M. mucogenicum* and related strains. The TY81 strain satisfied three of four conserved signature indels of Mycolicibacterium [9]. The three conserved signatures were a 5 aa insertion of GDAQS at positions 197–201 in the LacI family transcriptional regulator gene, a 1 aa insertion of proline at position 60 in the CDP-diacylglycerol-glycerol-3-phosphate 3-phosphatidyl transferase gene, and 1 aa deletion at position 128 in the CDP-diacylglycerol-serine O-phosphatidyl transferase gene. The protein of Cyclase gene (Accession number; WP_066808156) was not detected by homology search using protein-protein Basic Local Alignment Search Tool (BLASTp) with the threshold of 90% of similarity.

**Table 1:** ANI calculation

Species name	Strain	Refseq accession number	Refseq category	ANI (%)
*Mycolicibacterium mucogenicum*	CSUR P2099	GCF_001291445.1	Representative genome	93.3
*Mycolicibacterium phocaicum*	JCM 15301^T^	GCF_010731115.1	Representative genome	92.7
*Mycolicibacterium aubagnense*	JCM 15296^T^	GCF_010730955.1	Representative genome	88.4
*Mycolicibacterium houstonense*	ATCC 49403^T^	GCF_900078665.2	Representative genome	80.5
*Mycolicibacterium senegalense*	CK2	GCF_001021425.1	None	80.4
*Mycobacterium fortuitum*	CT6	GCF_001307545.1	Representative genome	80.3
*Mycobacteroides abscessus*	ATCC 19977^T^	GCF_000069185.1	Reference genome	78.2

Moreover, we performed additional examination, concerning 16S rRNA phylogeny may not distinguish between closely related species [10]. A comparison was made among TY81, the six strains of *M. mucogenicum* and the type strains of *M. phocaicum* and *M. aubagnense* by calculating the ANI values and constructing the phylogenetic tree based on core genomes consisting of 455 genes (Supplementary Fig. 1). The results from both analyses were consistent that all strains belonging to *M. mucogenicum* group are distinct from TY81. Note that the 4 of 6 strains of *M. mucogenicum* were closer to *M. phocaicum* as seen in Behra et al.^10^. Considering the genetic characteristics described above, we suggest that the TY81 strain is a novel species. The scientific name proposed for this species is *Mycolicibacterium toneyamachuris* sp. nov. with RIMD 1333001^T^ as the type strain. The bacterium was named ‘*Mycobacterium toneyamachuris*’ after the place where it was discovered.

*M. toneyamachuris* is a gram-positive, acid-fast, aerobic, anaerobic, non-pigmented and non-motile bacillus. Colonies were grown on 2% Ogawa-medium, Tryptic Soy Agar (TSA), and 5% Sheep Blood Agar and appeared greyish without pigmentation (Supplementary Fig. 2). Growth was observed within 7 days at 25, 30, and 37 °C temperature with optimal growth at 37 °C.

Currently, the treatment of NTM-PD caused by rare species/subspecies is a process of trial and error. It often starts with the drug regimen clinically used for closely related species/subspecies, which is modified by in vitro drug sensitivity test results (Table 2). Because the closest species/subspecies to our strain was *M. mucogenicum*, an RGM for which macrolides, quinolones, and amikacin are the most commonly used drugs [6], we started combination therapy with clarithromycin (CAM) 800 mg/day, moxifloxacin (MFLX) 400 mg/day, amikacin (AMK) 400 mg/day, and imipenem/cilastatin (IPM/CS) 1500 mg/day. Her clinical symptoms and chest computed tomography 2 months after starting chemotherapy showed significant improvement (Fig. 1).

**Table 2:** Antimicrobial drug susceptibility for *Mycobacterium toneyamachuris*

Drug	Susceptibility	MIC (μg/ml)
CAM (3 days)		0.5
CAM	S	0.5
AZM (3 days)		2
AZM		4
CFX	S	16
IPM	S	1
MEPM	S	4
FRPM		8
AMK	S	4
TOB	R	16
MINO	R	> 16
DOXY	R	> 16
LZD	S	≤ 4
MFLX	I	2
CPFX	R	16
LVFX		4
ST	S	≤ 2/38

*S* Susceptible, *I* Intermediate, *R* Resistant

## Discussion and conclusion

This novel mycobacterium has two different characteristics from the closest species [6]. First, *M. mucogenicum* tended to cause catheter-related bacteremia but little pulmonary disease. M. mucogenicum pulmonary disease are reported to be rare and mainly occurs in immunocompromised patients. The patient was immunocompetent. However, we could not exclude the possibilities that treatment with inhaled corticosteroid made her susceptible to the NTM-PD [11]. Second, our strain showed different drug susceptibility to tetracyclines and quinolones compared with *M. mucogenicum,* an RGM that is relatively susceptible to multidrug treatment. In general, *M. mucogenicum* is susceptible for amikacin, cefoxitin, clarithromycin, carbapenems, fluoroquinolones and tetracyclines, although, tetracycline resistant straines are detected in about 20 to 40% of patients. Our strain showed resistance to minocycline, doxycycline and ciprofloxacin.

NTM include mycobacteria other than *M. tuberculosis* and *M. leprae* and consist of approximately 200 NTM species that are potentially pathogenic [12, 13]. Because conventional methods have only identified a small number of NTM species, NTM cultured from respiratory samples are sometimes unidentifiable. However, we should strive to identify pathogenic NTM, since NTM have various pathogenicity and prognosis at species/subspecies level [1, 14, 15]. Actually, *M. toneyamachuris* have distinct characteristics from even the closest species; *M. mucogenicum*. Although one strain does not necessarily represent characteristic of its species, our case indicates importance of accurate identification and usefulness of genomic sequencing.

With the advancement of identification techniques, increasing numbers of novel bacterial species that are potentially pathogenic will be identified in human samples. The identification and classification of undiscovered pathogenic NTM species may enhance our understanding of mycobacterial diseases. In the future, data-sets of clinical phenotypes and bacterial DNA sequences might help elucidate the pathogenesis of NTM.

## Supplementary Information

**Additional file 1 Supplementary Figure 1.** Whole genomic comparison of M. toneyamachurisand M. mucogenicumgroup. A) Mutual similarity using average nucleotide identity. B) Phylogenetic tree generated by core genome consisting of 455 genes. **Supplementary Figure 2**. The colony formation of TY81 on Tryptic Soy Agar at 30 °C at day 7. Scale bar indicates 2 mm.

## Data Availability

The datasets supporting the conclusions of this article are included within the article.

## Abbreviations

AMK: Amikacin
ANI: Average nucleotide identity
ATS/IDSA: American Thoracic Society/Infectious Diseases Society of America
BLASTp: Protein-protein Basic Local Alignment Search Tool
CAM: Clarithromycin
CT: Computed tomography
DDH: DNA − DNA hybridization assay
IPM/CS: Imipenem/cilastatin
MFLX: Moxifloxacin
*M. leprae*: *Mycobacterium leprae*TSA: Tryptic Soy Agar*M. mucogenicum*: *Mycolicibacterium mucogenicum*
*M. toneyamachuris*: *Mycobacterium toneyamachuris*
*M. tuberculosis*: *Mycobacterium tuberculosis*
NTM: Non-tuberculous mycobacterial
NTM-PD: Non-tuberculous mycobacterial pulmonary disease
RGM: Rapid growing mycobacterium
