# Supplementary material for: Pulmonary disease caused by a newly identified mycobacterium: Mycolicibacterium toneyamachuris: a case report
Source: BMC Infect Dis. 2020 Nov 25;20:888. doi: 10.1186/s12879-020-05626-y (PMC7690136; doi:10.1186/s12879-020-05626-y)

Supplementary Figure 1. Whole genomic comparison of M. toneyamachuris and M. mucogenicum group. A) Mutual similarity using average nucleotide identity. B) Phylogenetic tree generated by core genome consisting of 455 genes.

A

	M. toneyamachuris TY81	*M. mucogenicum* 1127319.6	*M. mucogenicum* CSUR P2099	*M. mucogenicum* CCH12-A2	*M. mucogenicum* CCH10-A2	*M. mucogenicum* 1199456.5	*M. mucogenicum* 24AIII	*M. phocaicum* JCM 15301	*M. aubagnense* JCM 15296
M. toneyamachuris TY81	100	93.4	93.3	93.3	93.1	93.4	93.6	92.7	88.5
M. mucogenicum 1127319.6	93.5	100	96.9	92.2	92.1	92.3	92.3	91.9	88.4
M. mucogenicum CSURP 2099	93.3	96.8	100	92.1	92.1	92.2	92.3	91.8	88.5
M. mucogenicum CCH12-A2	93.4	92.2	92.2	100	99.9	97.0	97.0	95.4	88.6
M. mucogenicum CCH10-A2	93.3	92.2	92.2	99.9	100	97.0	96.9	95.3	88.6
M. mucogenicum 1199456.5	93.4	92.4	92.3	97.0	96.9	100	96.8	95.5	88.6
M. mucogenicum 24AIII	93.5	92.2	92.3	97.0	96.8	96.9	100	95.4	88.6
M. phocaicum JCM 15301	92.8	91.8	91.8	95.4	95.3	95.5	95.4	100	88.6
M. aubagnense JCM 15296	88.6	88.6	88.6	88.7	88.6	88.6	88.8	88.6	100

B

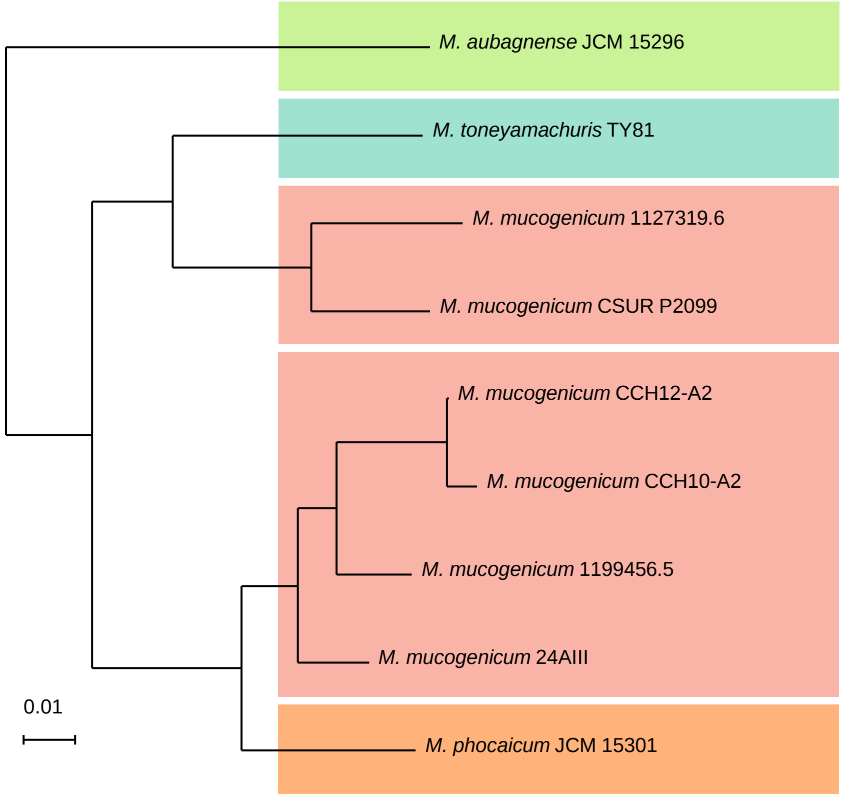

Supplementary Figure 2. The colony formation of TY81 on Tryptic Soy Agar at 30 ° C at day 7.
Scale bar indicates 2 mm.

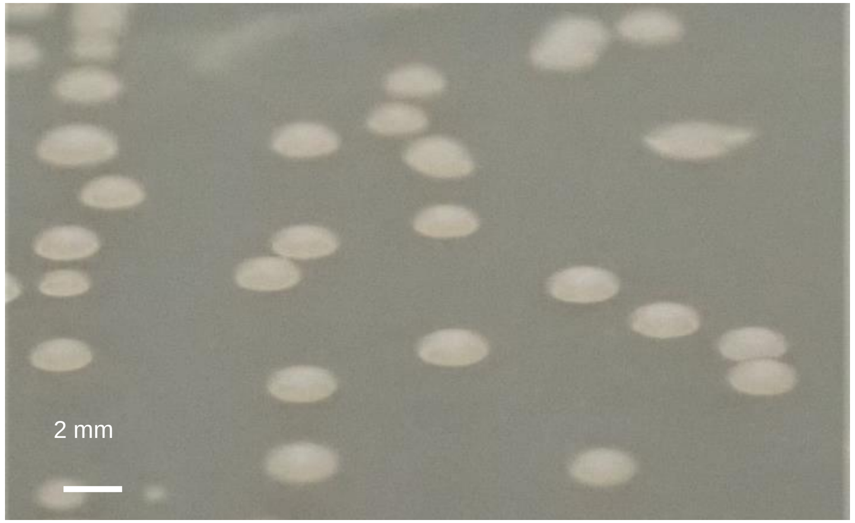

Supplement: Supplementary file 1 — Additional file 1 Supplementary Figure 1. Whole genomic comparison of M. toneyamachurisand M. mucogenicumgroup. A) Mutual similarity using average nucleotide identity. B) Phylogenetic tree generated by core genome consisting of 455 genes. Supplementary Figure 2. The colony formation of TY81 on Tryptic Soy Agar at 30 °C at day 7. Scale bar indicates 2 mm. [file 12879_2020_5626_MOESM1_ESM.pdf]
